# Examining the Effect of Consuming C_8_ Medium-Chain Triglyceride Oil for 14 Days on Markers of NLRP3 Activation in Healthy Humans

**DOI:** 10.1155/2022/7672759

**Published:** 2022-04-06

**Authors:** Helena Neudorf, Garett Jackson, Jonathan P. Little

**Affiliations:** School of Health and Exercise Sciences, University of British Columbia, Okanagan Campus, Kelowna V1V 1V7, Canada

## Abstract

Chronic, low-grade inflammation is associated with the development of numerous diseases and is mediated in part by overactivation of the NLRP3 inflammasome. The ketone body beta-hydroxybutyrate (*β*HB) suppresses the NLRP3 inflammasome and alters intracellular signalling pathways in vitro and in animal models; however, this effect has not yet been shown in vivo in humans. The purpose of this single-arm pilot trial was to determine if consuming 15 mL of C_8_ medium-chain triglyceride (trioctanoin; MCT) oil, which induces mild elevation of *β*HB, twice daily (30 mL total) for 14 days would suppress markers of NLRP3 inflammasome activation in young, healthy humans while following their habitual diet. Consuming a single dose of 15 mL of C_8_ MCT oil significantly raised blood *β*HB from fasting at 60 minutes and 120 minutes post ingestion (both *P* < 0.05). However, consumption of C_8_ MCT oil for 14 days did not impact markers of monocyte NLRP3 inflammasome activation compared to baseline. Specifically, caspase-1 activation and secretion of its downstream product interleukin (IL)-1*β* were unchanged following 14 days of C_8_ MCT oil supplementation when measured in unstimulated and LPS-stimulated whole blood cultures (all *P* > 0.05). Acetylation of histone H3 on the lysine residue 9 was unchanged (*P* < 0.05) and acetylation of lysine residue 14 was decreased (*P* < 0.05) following 14 days of supplementation. Thus, adding twice daily C_8_ MCT oil supplementation to the habitual diet of young, healthy humans does not appear to suppress NLRP3 inflammasome activation.

## 1. Introduction

Chronic low-grade inflammation, characterized in part by high levels of inflammatory cytokines in circulation, has been widely implicated in the development of a number of chronic diseases (e.g., type 2 diabetes and atherosclerosis) [1–3] and thus identification of strategies to reduce such inflammatory signalling is of great interest. The ketone body beta-hydroxybutyrate (*β*HB) is one such strategy that has been shown to have promising anti-inflammatory and antioxidant signalling properties in animal and in vitro models [4–9]. Ketones are produced endogenously in response to ketogenic diets, caloric restriction, and fasting [10], with *β*HB being the most abundant of the ketone bodies produced [11]. *β*HB, but not other ketone bodies, has been found to dose-dependently attenuate markers of activation of the nucleotide-binding oligomerization domain (NOD)-like receptor pyrin domain containing 3 (NLRP3) inflammasome, an important inflammatory mediator and sensor of nutrient overload, thereby reducing secretion of the proinflammatory cytokine interleukin (IL)-1*β* [4]. Additionally, *β*HB has been found to reduce markers of inflammation which are associated with gout [5] as well as increasing expression of antioxidant genes [4, 6–9], effects attributed to *β*HB acting as a class I histone deacetylase inhibitor [6]. Thus, *β*HB holds promise as a nonpharmaceutical means to suppress inflammatory signalling.

Various dietary manipulations, including fasting, very-low carbohydrate high-fat ketogenic diets, and supplements that contain ketones or their precursors can raise plasma *β*HB in humans. Medium-chain triglyceride (MCT) oil is one such ketogenic supplement that has been demonstrated to elevate blood *β*HB to levels achieved in mild ketosis. MCTs are defined as fatty acids that are between 6 and 10 carbons in length (C_12_ can be characterized as a medium- or longer chain fatty acid), with C_8_ having the greatest ketogenic effect [12]. Upon consumption, MCTs are absorbed quickly and the liberated fatty acids are either oxidized in the liver for energy or metabolized into *β*HB by the liver such that circulating *β*HB increases. Feeding of MCTs to mice with high-fat diet-induced obesity attenuated circulating IL-6 levels and activation of hepatic nuclear factor kappa-light-chain-enhancer of activated B cells (NF-*κ*B) and p38 mitogen-activated protein kinase (MAPK), while enhancing IL-10 secretion compared to controls [13]. Similarly, 3 weeks of feeding of MCTs to piglets prior to a lipopolysaccharide (LPS)-challenge resulted in attenuated tumour necrosis factor (TNF) *α*, IL-6, and IL-1*β* levels, reduced toll-like receptor (TLR) 4, NOD1, and p38 MAPK expression and protected against liver injury [14]. In a separate study, three weeks of feeding MCTs to piglets also protected against intestinal injury (via intraperitoneal injection of LPS) and suppressed local expression of TLR4, TNF*α*, NF-*κ*B, NOD2, and receptor-interacting serine/threonine-protein kinase (RIPK) 2 [15]. In neonates with sepsis, nutritional supplementation with a lipid emulsion consisting of MCT, olive, fish, and soy oil for 7 days led to increased soluble intercellular adhesion molecule 1 (sICAM1) and leukocyte *β*2 integrin, which are both associated with improved sepsis outcomes [16]. Thus, MCTs appear to have unique immunomodulatory signalling properties, potentially via the mild ketosis induced by their consumption and thus could be one dietary strategy to alter inflammatory signalling in immune cells. To our knowledge, the specific effects of MCTs on key inflammatory signalling pathways such as the NLRP3 inflammasome in humans have not been explored.

Therefore, the purpose of this pilot study was to explore if consumption of MCT oil twice daily for 14 days would attenuate markers of LPS-stimulated NLRP3 inflammasome activation in healthy humans following their habitual diet. Gaining an understanding of the potential immunomodulatory effects of MCT oil consumption may help to inform future nonpharmaceutical therapeutic approaches to suppressing chronic low-grade inflammation.

## 2. Materials and Methods

### 2.1. Study Approval

We conducted a single-arm pilot study to investigate the effect of short-term elevation of *β*HB by C_8_ MCT oil supplementation on markers of NLRP3 inflammasome activation and histone H3 acetylation in young, healthy individuals. This pilot study was approved by the University of British Columbia Clinical Research Ethics Board (ID H17-03258) and was registered as “The Effect of Raising Ketones Directly with MCT Oil on Inflammation in Healthy Young Adults” (ClinicalTrials.gov identifier NCT03460444).

### 2.2. Participants

Sixteen participants (6 females; 10 males) were recruited by poster and word-of-mouth at the University of British Columbia Okanagan campus and around Kelowna. One participant dropped out of the study due to gastrointestinal upset in response to the C_8_ MCT oil supplement such that data from *N* = 15 are reported here (*N* = 6 females; *N* = 9 males). Participants were included if they were 18–30 years of age, had a body mass index (BMI) of 18.5 to 29.9 kg/m^2^ (i.e., normal to overweight), were not taking any anti-inflammatory medication, had not been following a ketogenic diet or intermittent fasting program within the previous three months, were free of metabolic disease, and did not have a history of inflammatory disorders. All participants provided written informed consent during the initial screening and familiarization visit.

### 2.3. Study Design

**Figure 1:**
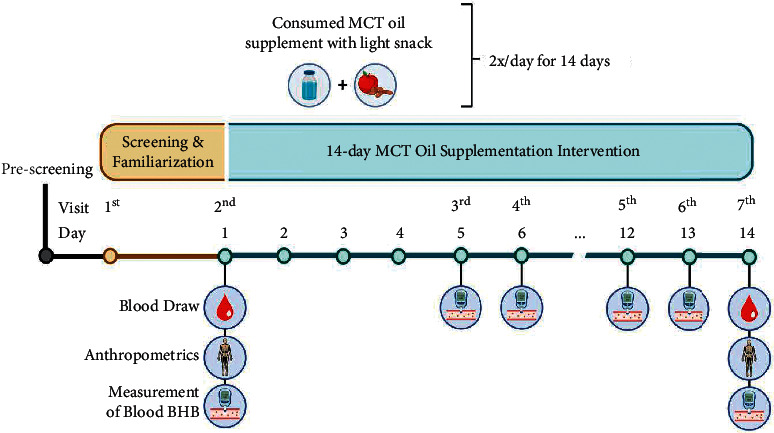
Depiction of the study design. Participants consumed one dose of MCT oil twice daily for 14 days. Markers of NLRP3 inflammasome activation were quantified on day 1 (Baseline) and day 14 (Post).

The study design is represented in Figure 1. Participants attended an initial screening visit, during which they reviewed the consent form, had an opportunity to ask questions, and were familiarized with the procedures if they provided written informed consent. Participants completed a three-day dietary recall and were asked to maintain and track their current diet throughout the 14-day intervention. Female participants began the intervention during the follicular phase (days 4–7) of their menstrual cycle. Participants arrived to their first testing visit (Pre) in the morning having fasted for at least the ten hours prior. Age, height, weight, and waist circumference were measured. Fasting blood *β*HB levels were also measured by finger prick (FreeStyle Precision Neo, Abbott Laboratories), and 12 mL of blood was collected from the antecubital vein was collected by a trained phlebotomist for assessment of indices of inflammation, histone H3 acetylation, insulin, and glucose. This visit was replicated at the end of the 14-day trial (Post). Participants consumed 15 mL of C_8_ MCT oil supplement twice per day 2–3 hours following breakfast and lunch. The supplement was consumed with a light snack as recommended by the manufacturer in order to reduce the chances of adverse gastrointestinal reactions. Finger prick *β*HB levels were collected at one and two hours following consumption of the C_8_ MCT oil supplement a total of eight times throughout the 14-day trial (twice per day on visits 3–6) to verify that the C_8_ MCT oil supplement was raising blood *β*HB levels.

### 2.4. C_8_ MCT Oil Supplement

The C_8_ MCT oil supplement (KetoMCT, Lifesense® Products) was made up of 97–99% C_8_ caprylic (octanoic) acid triglycerides, with the remainder being C_10_ (decanoic) triglycerides, with the glycerol and fatty acid components derived from coconut oil. One serving (15 mL) contained 15 g of MCTs. As mentioned previously, MCT oil supplements can induce gastrointestinal distress in some individuals; therefore, in order to minimize the likelihood of adverse events, the dose was gradually increased from 5 mL twice per day on days 1-2 to 10 mL twice per day on days 3–4, to 15 mL twice per day for the remainder of the study. Prior to beginning the trial, participants completed a three-day dietary recall. Participants then met with a researcher to review their diet log, and were instructed to maintain their habitual diet as closely as possible while aiming for a macronutrient distribution of 40–50% of energy from carbohydrates, 30–40% of energy from fats (excluding MCT oil supplements), and 20–25% of energy from proteins. Diet was otherwise not modified or controlled throughout the 14 days of supplementation in order to determine whether adding MCT oil to participants' habitual dietary pattern influenced immune markers. In order to confirm compliance with the macronutrient targets, we randomly selected three days of food records completed during the intervention for each participant for analyses (using MyFitnessPal).

### 2.5. Quantification of NLRP3 Inflammasome Activation

**Figure 2:**
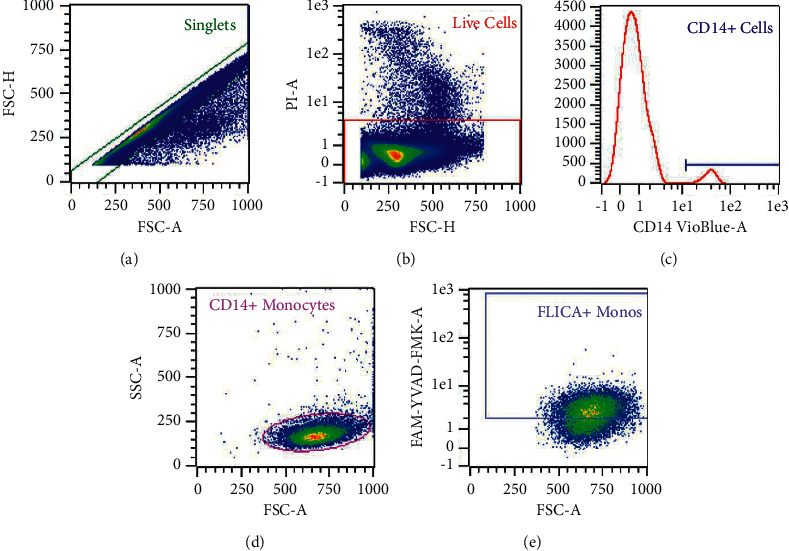
Hierarchical gating strategy for quantification of activated caspase-1 by flow cytometry on a representative sample. (a) Singlets were selected. (b) Live cells were selected for negative staining for propidium iodide (PI). (c, d) Monocytes were selected by positive staining for CD14 and scatter characteristics. (e) Monocytes displaying positive staining for FAM-FLICA (representative of expression of activated caspase-1) were quantified by median fluorescence intensity and percent positive.

NLRP3 inflammasome activation was quantified by measuring both activated caspase-1 and secreted IL-1*β* in whole blood culture cultures using the fasting samples at baseline and 14 days. Caspase-1 was measured by flow cytometry using the carboxyfluorescein fluorochrome inhibitor of caspases (FAM-FLICA®) Caspase-1 Assay Kit containing the fluorescent inhibitor probe FAM-YVAD-FMK (FAM-FLICA®; ImmunoChemistry Technologies, Bloomington, MN, USA). This protocol has previously been described in detail elsewhere [17]. Briefly, fresh ethylenediaminetetraacetic acid (EDTA) whole blood was treated with LPS (10 ng/mL in culture) or an equivalent volume of media (control) and cultured for 60 minutes at 37°C and 5% CO_2_ in the dark. FAM-FLICA was added (1X in culture) and samples were incubated for a further 50 minutes 37°C and 5% CO_2_ in the dark. Samples were then incubated with cluster of differentiation (CD) 14 for 10 minutes, red blood cells were lysed, and samples were washed twice in apoptosis wash buffer. Immediately prior to data acquisition by flow cytometry (MACSQuant® Analyzer, Miltenyi Biotec, Bergisch Gladbach, Germany), propidium iodide (PI) was added for dead cell exclusion. Gates were set by fluorescence minus one (FMO) controls. The hierarchical gating strategy is presented in Figure 2.

IL-1*β* from culture supernatants was measured by Human IL-1 beta/IL-1F2 DuoSet enzyme-linked immunosorbent assay (ELISA) (R&D Systems) per the manufacturer's instructions. The lower limit of detection was ∼10 pg/mL. Fresh EDTA whole blood was treated with LPS (10 ng/mL in culture) or media (control) and cultured for 2 hours at 37°C and 5% CO_2_ in the dark. Cultures were then centrifuged and the supernatants were pipetted off and frozen at −80°C for later batch analysis.

### 2.6. Quantification of Histone H3 Acetylation

**Figure 3:**
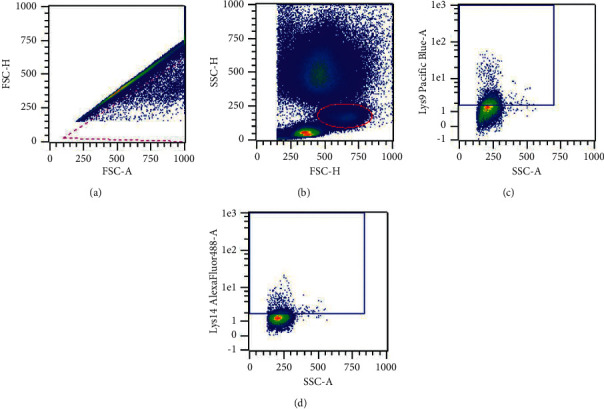
Hierarchical gating strategy for quantification of acetylated histone H3 on lysine residues 9 and 14 on a representative sample. (a) Singlets were included by negative selection. (b) Monocytes were selected by characteristics scatter profile. (c, d) Staining for acetylation of lysine residues 9 and 14 was quantified by the median fluorescence intensity and percent staining positive on monocytes.

Acetylation of histone H3 lysine residues 9 (Lys9) and 14 (Lys14) was quantified by flow cytometry. Fresh EDTA whole blood was collected and incubated sequentially with FcR blocking reagent and CD14 (Miltenyi Biotec) for ten minutes at 4°C in the dark each. Red blood cells were lysed and washed once. Samples were then fixed in 2% formaldehyde for 15 minutes at room temperature in the dark, washed in PBS, then permeabilized in 0.05% Tween 20 for five minutes at room temperature in the dark. Samples were stained with anti-Lys9 Pacific Blue, anti-Lys14 Alexa-Fluor 488, and incubation buffer (0.5% bovine serum albumin (BSA) in phosphate-buffered saline (PBS)) for 30 minutes, then washed twice in PBS, and analyzed immediately. Gates were set by FMO controls. The hierarchical gating strategy is presented in Figure 3.

### 2.7. Gastrointestinal Distress

Participants were asked to complete a questionnaire assessing symptoms of gastrointestinal distress (belching, bloating, cramping, nausea, and urge to vomit) using a 6-point scale (scores of 0 to 5) at 60 and 120 minutes post supplementation on visits 3–6 (i.e., on days 5, 6, 12, and 13 of the intervention at the same time as finger stick blood *β*HB was measured). A score of zero was representative of the complete absence of the symptom while a score of five was representative of the worst imaginable manifestation of the symptom. The median of responses across all four measurement days at both 60 and 120 minutes are reported as descriptive data.

### 2.8. Fasting Glucose and Insulin

Glucose was quantified by a glucose oxidase activity assay. Briefly, glucose standards were diluted by serial dilution and standards, controls (glucose, Sigma-Aldrich), and samples were aliquoted into a 96-well plate in duplicate. Glucose oxidase (Infinity Glucose Oxidase, Thermo Scientific, Altham, MA, USA) was then aliquoted into each well. The plate was then shaken for 30 seconds and incubated for 10 minutes at 37°C. The plate was read on a plate reader (BioRad iMark, Herculus, CA, USA) with a 540 nm filter.

Insulin was quantified by enzyme-linked immunosorbent assay (ELISA, Mercodia, Uppsala, Sweden) per the instructions on the aforementioned plate reader.

### 2.9. Statistics

All statistical analyses were performed using *R* [18]. A sample size calculation was conducted using means and standard deviations for monocyte caspase-1 in healthy, young adults from previous research conducted in our lab [19] and found that a sample size of 14 was powered at 90% to detect a moderate effect size of 0.5 with a two-tailed *P* value of 0.05. Sixteen participants were recruited to account for missing samples and dropouts. Data were analyzed using a linear mixed effects model using the lme4 package in *R* with fixed effects for time point (pre or post) and culture condition (LPS-stimulated or unstimulated basal), and a random intercept for participant [20]. Data were assessed visually using normal probability plots and residuals vs. fitted values plots. Model residuals that did not meet assumptions were log transformed, and effect estimates and 95% confidence intervals were back transformed using the emmeans package [21]. Presence of influential data points was assessed by calculating Cooks distance using the influence.ME package [22] and excluded if the Cook's distance value exceeded the cutoff of 4/*n* [23]. *β*HB values that were below the lower limit of detection (LLOD; 0.1 mM) were imputed using the method recommended by Canales et al. [24]: $\text{LLOD}/\sqrt{2}$. Calories consumed at baseline and during the intervention were compared using a paired Student's *T*-test. All data are presented as estimated marginal means with 95% confidence interval unless stated otherwise.

## 3. Results and Discussion

### 3.1. Participants

Participant anthropometric characteristics were unchanged from baseline at the end of the 14-day C_8_ MCT oil supplementation (Table 1). Based on baseline 3-day diet records, participants consumed an average of 2265 (709) kcal per day, with 21% (8) of their energy coming from protein, 42% (9) of energy from carbohydrates, and 37% (9) of energy from fats (data are means (SD)). Based on self-report diet records, during the intervention average calories (excluding C_8_ MCT oil) were 2202 (663) kcal per day (*P*=0.802 vs. baseline), with 23% (11) of energy from protein, 41% (9) of energy from carbohydrates, and 36% (33) of energy from fat. The C_8_ MCT oil added approximately 270 kcal with 30 g of fat per day, such that including the supplement, average calories consumed were 2472 kcal (*P*=0.183 vs. baseline), with 21% (10) of energy from protein, 36% (8) of energy from carbohydrates, and 44% (7) of energy from fats.

### 3.2. Blood Beta-Hydroxybutyrate

**Figure 4:**
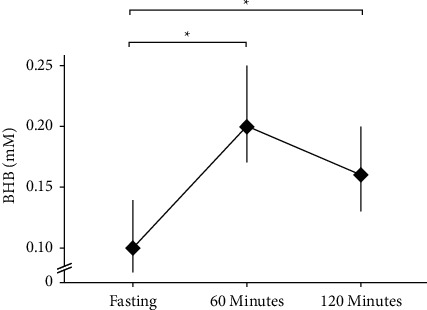
Beta-hydroxybutyrate (*β*HB) measured from capillary blood. *β*HB measured at fasting, and 60, and 120 minutes after one dose of C8 MCT oil throughout the 14-day trial. Data are estimated marginal means from a linear mixed effects model plotted with a 95% confidence interval. *β*HB was significantly higher at 60 and 120 minutes compared to fasting values. ^*∗*^*P* < 0.05; *N* = 15.

Blood *β*HB was measured at fasted baseline and post intervention, as well as 60- and 120-minutes following ingestion of a single dose of C_8_ MCT oil. Blood *β*HB was significantly raised from fasting values (0.10 mM, CI95 (0.08, 0.14)) at both 60 minutes (0.2 mM, CI95 (0.17, 0.25); *P* < 0.001) and 120 minutes (0.16 mM, CI95 (0.13, 0.20); *P* < 0.001) following a single dose of C_8_ MCT oil (Figure 4).

### 3.3. Activated Caspase-1

**Figure 5:**
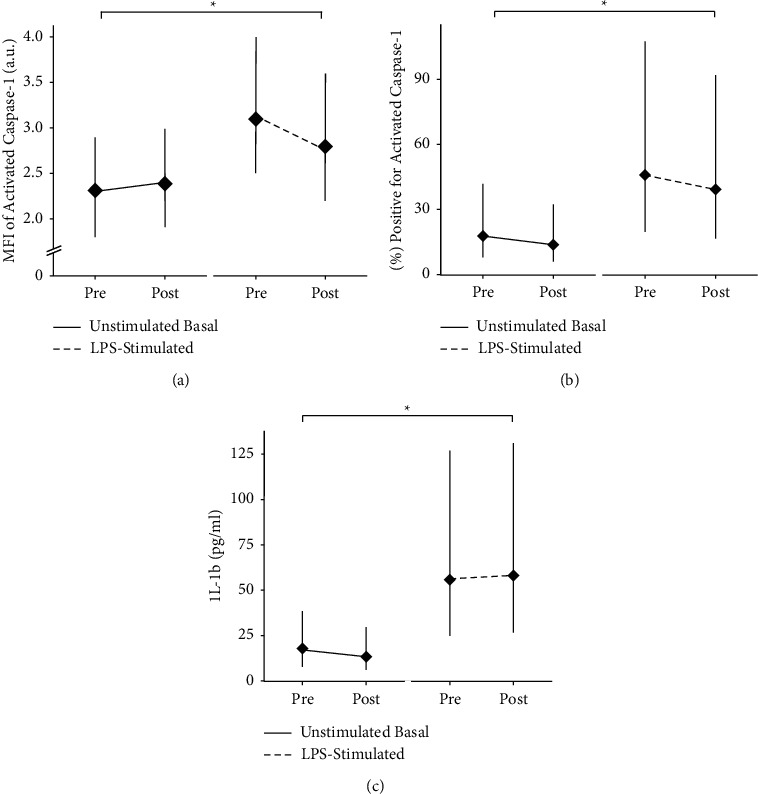
Quantification of LPS-stimulated or unstimulated basal activated caspase-1 of CD14^+^ monocytes by flow cytometry Pre- and Post-MCT oil supplementation for 14 days. Premeasurements were obtained on the first day (day 1) of the intervention and postmeasurements were obtained on the last (day 14) day of the intervention. (a) Median fluorescence intensity (MFI) of activated caspase-1 measured in arbitrary fluorescence units (a.u.). (b) Percent of cells staining positive for activated caspase-1. (c) Secreted IL-1*β* into LPS-stimulated and unstimulated whole blood cultures. Data are estimated marginal means from a linear mixed effects model plotted with a 95% confidence interval. ^*∗*^Simple effect of culture condition; *P*=0.600. Caspase-1; *N* = 15; IL-1*β*; *N* = 14.

Estimated marginal means and *P* values from the linear mixed effects model for activated caspase-1 are reported in Supplemental Table 1. As expected, median fluorescence intensity (MFI) of unstimulated basal caspase-1 activation was significantly lower compared to LPS-stimulated caspase-1 activation in CD14+ monocytes (main effect of condition; *P*=0.003; Figure 5(a)), but there was no difference in caspase-1 activation after 14 days of C_8_ MCT oil supplementation when compared to baseline. Similarly, the percent of unstimulated CD14^+^ monocytes staining positive for basal activated caspase-1 was significantly lower compared to the LPS-stimulated condition (main effect of condition; *P* < 0.001; Figure 5(b)), but the percent staining positive for activated caspase-1 was not different between timepoints and there was no interaction between timepoint and culture condition.

### 3.4. Secreted IL-1*β*

Estimated marginal means and *P* values from the linear mixed effects model for IL-1*β* secreted into culture are reported in Supplemental Table 1. Basal IL-1*β* secretion from unstimulated cultures was significantly lower compared to LPS-stimulated IL-1*β* secretion (main effect of condition; *P* < 0.001; Figure 5(c)). However, C_8_ MCT oil supplementation did not have an effect on either basal or LPS-stimulated IL-1*β* secretion compared to baseline (*P* > 0.05).

### 3.5. Monocyte Histone H3 Acetylation

Acetylation of monocyte histone H3 on lysine residues 9 (Lys9) and 14 (Lys14) was quantified at baseline and following 14 days of supplementation with C_8_ MCT oil. Full results are reported in Table 2. No difference was found in the percent of cells staining positive for acetylated Lys9 (*P* > 0.05), and the MFI of acetylated Lys9 was not statistically different between time points. Similarly, no difference was found in the percent of cells staining positive for Lys14 (*P* > 0.05). However, the MFI of acetylated Lys14 was significantly lower following 14 days of supplementation (*P* > 0.05).

### 3.6. Fasting Insulin and Glucose

Plasma insulin at fasted baseline was 105.6 pmol/L, 95% CI (66.8, 166.7). After 14 days of C_8_ MCT supplementation, fasting insulin was 124 pmol/L, 95% CI (78.5, 195.9), with no significant difference between these two time points (*P*=0.612; *N* = 14).

Plasma glucose at fasted baseline was 5.7 mM, 95% CI (5.4, 6.0). After 14 days of C_8_ MCT supplementation, fasted glucose was 5.8 mM, 95% CI (5.5, 6.0) with no significant difference between these two time points (*P*=0.600; *N* = 15).

### 3.7. Gastrointestinal Distress Scores

GI distress scores for belching, bloating, cramping, nausea, and urge to vomit were assessed 60 and 120 minutes on four separate days throughout the intervention. There were wide range of scores with some participants reporting no symptoms (i.e., scores of 0) and some reporting severe symptoms (i.e., scores of 5). The median score each for belching, bloating, and cramping was 0 at both 60 and 120 minutes and ranged from scores of 0 to 5 (*N* = 11). The median score each for nausea and urge to vomit was 0 at both 60 and 120 minutes and ranged from score of 0 to 4 (*N* = 11).

## 4. Discussion

Direct elevation of *β*HB has been shown in several animal and in vitro studies to suppress activation of the NLRP3 inflammasome in response to various inflammatory stimuli. However, whether raising *β*HB through dietary means in humans can alter inflammatory signalling in immune cells has not been confirmed. Here, we report that mild elevation of circulating *β*HB through 14 days of C_8_ MCT oil supplementation does not appear to alter basal or LPS-stimulated monocyte NLRP3 inflammasome activation in healthy, young male and female participants who continued to follow their habitual diet.

### 4.1. C_8_ MCT Oil Supplementation Does Not Suppress Basal NLRP3 Activation

We assessed the effect of C_8_ MCT oil supplementation on markers of NLRP3 inflammasome activation in the basal state. MFI of activated caspase-1, percent of cells staining positive for activated caspase-1, and IL-1*β* secretion from whole blood cultures were unchanged in the basal state (Figures 5(a)–5(c)). This complements previous results from our group in which we reported that acute elevation of blood *β*HB by consumption of a single dose of ketone monoester supplement did not alter markers of NLRP3 inflammasome activation in the basal state in healthy individuals under similar conditions [17]. It has been reported that basal NLRP3 inflammasome activation is increased in individuals with obesity and type 2 diabetes [25] and thus, it is plausible that we did not see an effect of C_8_ MCT oil supplementation due to our participants being young and healthy; a certain level of innate immune activation must be present in order for adequate protection of the host. However, in contradiction to this theory, our group has also found no effect of both acute and short-term (14 days) elevated blood *β*HB by ketone monoester (an agent known to increase circulating *β*HB more robustly than C_8_ MCT oil) on NLRP3 inflammasome activation in the basal state in individuals with obesity [26, 27]. If a critical threshold of basal or chronic low-grade inflammation is necessary in order for *β*HB to have an anti-inflammatory effect, it is unclear at what level this threshold may be. Thus, 14 days of supplementation with C_8_ MCT oil did not alter markers of NLRP3 inflammasome activation in the basal state under the present experimental conditions.

### 4.2. C_8_ MCT Oil Supplementation Does Not Suppress LPS-Stimulated NLRP3 Activation

It was also of interest to quantify the effect of C_8_ MCT oil supplementation on markers of NLRP3 inflammasome activation in response to an immune challenge. MFI of activated caspase-1 and the percent of cells staining positive for activated caspase-1 were unchanged (Figures 5(a)–5(c)), and IL-1*β* secretion into LPS-stimulated cultures was unchanged by C_8_ MCT oil supplementation. This is in contradiction to previous animal and in vitro works showing a suppression of LPS-mediated NLRP3 inflammasome activation [4–9]. These studies tested the effectiveness of *β*HB using concentrations of between 2 mM and 20 mM, while C_8_ MCT oil supplementation in the present study only induced mild ketosis of ∼0.2 mM measured at 60 minutes after each single dose (Figure 4). These previous studies have not tested if such low elevations in *β*HB can alter NLRP3 inflammasome activation; however, it seems likely that the C_8_ MCT oil supplementation under the present experimental conditions is not able to raise *β*HB to levels high enough to induce measurable suppression of proinflammatory signalling. Zhang et al. reported that feeding pigs 4% MCTs (53% C_8_; 46% C_10_) for three weeks suppressed circulating TNF*α*, IL-1*β*, and IL-6 as well as hepatic expression of TLR4 and NOD1 and led to changes in lipid profile [14]. Thus, it is possible that MCTs mediate their anti-inflammatory effect through a means other than inducing mild ketosis.

### 4.3. Histone Acetylation

*β*HB has been shown to be a histone deacetylase inhibitor, which impacts expression of genes involved in anti-oxidant responses [6]. Therefore, as an exploratory aim we assessed whether C_8_ MCT oil supplementation, through raising blood *β*HB, might impact the acetylation status of histone H3. Results revealed no impact on the percent of monocytes staining positive for acetylated Lys9 or Lys14. Interestingly, the MFI of monocytes for Lys14 decreased following 14 days of C_8_ MCT oil supplementation, an effect that would appear opposite to the proposed actions of a histone deacetylase inhibitor. However, these data should be taken with caution as the risk for false positives is increased when analysing multiple exploratory outcomes, particularly in a single-arm pre-post pilot study.

### 4.4. GI Symptoms

Despite following a progressive increase in C_8_ MCT oil consumption, several participants still reported high GI discomfort scores in response to acute consumption, as evidenced by the range of values reported on the GI distress questionnaires. Given that 15 mL of C_8_ MCT oil only raised blood *β*HB to ∼0.2 mM and that this dose did appear to cause some GI discomfort in some participants, it seems unlikely that supplementing with C_8_ MCT oil is a feasible strategy if an individual's goal is to chronically raise blood *β*HB to appreciable levels.

### 4.5. Strengths and Limitations

To our knowledge, this pilot study was the first to test if C_8_ MCT oil supplementation can suppress inflammatory signalling through the NLRP3 inflammasome in humans. This study was strengthened by the use of high-throughput multicolour flow cytometry to determine basal and stimulated NLRP3 inflammasome activation and IL-1*β* secretion in human blood monocytes, thus measuring inflammation at the cellular/molecular level. This study was limited by the low levels of *β*HB experienced following C_8_ MCT oil consumption and the inability to precisely match each participant's exposure to *β*HB throughout. Additionally, this study was performed in free-living conditions over a relatively short duration and C_8_ MCT oil supplements were not added to the diet with strict isocaloric control. Acknowledging the limitations of self-report diet records, participants did appear to maintain their habitual diet across the 14 days and added C_8_ MCT oil, although there did appear to be a tendency for an increase in energy intake (and % energy from fats) when compared to prestudy measurements. Despite not detecting any change in body mass or waist circumference, without direct assessment of energy balance components, we cannot rule out the possibility that participants were in positive energy balance or experienced a compensatory increase in metabolic rate across the 14-day study. Nevertheless, the design provides insight into the effects of short-term consumption of C_8_ MCT oil in free-living conditions in individuals consuming a habitual diet.

### 4.6. Future Research

This study was a preliminary investigation into the effect of C_8_ MCT oil on cellular inflammatory parameters in healthy humans. Future research should investigate if directly elevating *β*HB by MCT oil or other supplements, on the background of different dietary patterns, can impact inflammatory signalling in various inflammation-mediated conditions such as obesity or type 2 diabetes. Additionally, different MCT lengths may have varying potencies [28] or signalling effects; therefore, future research should further investigate any differences that may exist. Furthermore, we investigated the immune-modulating effect of C_8_ MCT oil supplementation in the basal state and in response to a single ex vivo activating stimulus, LPS, which induces activation through TLR4 and has been used in previous animal studies [14, 15]. However, different stimuli induce immune cell activation through different signalling pathways and future research should investigate if the immune-modulating of MCT oil (or elevated *β*HB) could differ in response to different types of activators.

## 5. Conclusions

In summary, consuming 15 ml of C_8_ MCT oil twice daily for 14 days in young, healthy individuals induced mild ketosis but did not alter either basal or LPS-stimulated NLRP3 inflammasome activation. These findings will help inform future research on nonpharmaceutical therapeutic strategies for suppressing inflammation.

## Figures and Tables

**Table 1:** Anthropometrics at baseline (Pre-) and Post-MCT oil supplementation.

	Pre	Post	*P* value
Age (yrs)	23 (3)	—	—
Height (m)	1.74 (0.08)	1.74 (0.08)	—
Weight (kg)	75.9 (12.6)	76.6 (12.4)	0.88
BMI (kg/m^2^)	25.0 (2.5)	25.3 (2.5)	0.78
Waist circumference (cm)	83.1 (7.2)	82.6 (7.4)	0.84

Data are means (SD); *N* = 15.

**Table 2:** Acetylation of lysine residues 9 and 14 on monocyte histone H3.

	Estimated marginal means (95% CI)		*P* value from linear mixed effects model
	Pre	Post	Main effect of supplementation
Acetylated histone H3 lysine 9 (% positive)	12.6 (7.4, 21.3)	8.9 (5.3, 15.1)	0.201
Acetylated histone H3 lysine 9 (MFI; a.u.)^†^	4.3 (3.7, 5)	4.5 (3.8, 5.1)	0.247
Acetylation histone H3 lysine 14 (% positive)	1.2 (0.3, 4.4)	1.3 (0.4, 4.7)	0.677
Acetylated histone H3 lysine 14 (MFI; a.u.)	2.4 (2.1, 2.6)	2.2 (1.9, 2.4)	^*∗*^0.039

Data are estimated marginal means with 95% confidence intervals, and *P* values obtained from a linear mixed effects model. MFI, median fluorescence intensity. *N* = 12; ^†^*N* = 11.

## Data Availability

The data used to support the findings of this study will be made available upon reasonable request.

## Supplementary Materials

Supplementary Materials Supplemental Table 1: estimated marginal means for caspase-1 and IL-1*β* basally and from LPS-stimulated cultures.
