# Supplementary material for: Examining the Effect of Consuming C8 Medium-Chain Triglyceride Oil for 14 Days on Markers of NLRP3 Activation in Healthy Humans
Source: J Nutr Metab. 2022 Apr 6;2022:7672759. doi: 10.1155/2022/7672759 (PMC9007652; doi:10.1155/2022/7672759)
Supplement: Supplementary Materials — Supplemental Table 1: estimated marginal means for caspase-1 and IL-1β basally and from LPS-stimulated cultures. [file 7672759.f1.docx]

**Supplemental Table 1.** Estimated marginal means for caspase-1 and IL-1β basally and from LPS-stimulated cultures.

|  | **Estimated Marginal Means (95% CI)** | | | | **P-values from Linear Mixed Effects Model** | | |
| --- | --- | --- | --- | --- | --- | --- | --- |
|  | **LPS-Stimulated** | | **Unstimulated Basal** | | **Main Effect of Supplementation** | **Main Effect of Culture Condition** | **Interaction Effect** |
|  | **Pre** | **Post** | **Pre** | **Post** |  |  |  |
| **Activated Caspase-1 (MFI; a.u.)** | 3.1 (2.5, 4.0) | 2.8 (2.2, 3.6) | 2.3 (1.8, 2.9) | 2.4 (1.9, 3.0) | 0.308 | 0.003 | 0.425 |
| **Activated Caspase-1 (% Positive)** | 45.9 (19.6, 107.5) | 39.3 (16.8, 92.1) | 17.9 (7.7, 41.9) | 13.8 (5.9, 32.4) | 0.604 | <0.001 | 0.063 |
| **IL-1β (pg/ml)** | 56.5 (25, 127.6) | 58.2 (25.8, 131.5) | 17 (7.5, 38.5) | 13.2 (5.9, 29.8) | 0.840 | <0.001 | 0.187 |

Data are estimated marginal means with 95% confidence intervals and *P*-values obtained from a linear mixed effects model. MFI = median fluorescence intensity. Caspase-1, *N* = 15. IL-1β, *N* = 14.
